# Aberrant Lipid Metabolism in Hepatocellular Carcinoma Revealed by Liver Lipidomics

**DOI:** 10.3390/ijms18122550

**Published:** 2017-11-28

**Authors:** Zhao Li, Ming Guan, Yu Lin, Xiao Cui, Yangyang Zhang, Zhenwen Zhao, Jiye Zhu

**Affiliations:** 1 Department of Hepatobiliary Surgery, Peking University People’s Hospital, Beijing 100044, China; lizhao@bjmu.edu.cn (Z.L.); cx_2077@bjmu.edu.cn (X.C.); gandanwk@vip.sina.com (J.Z.); 2 Beijing National Laboratory for Molecular Sciences, Key Laboratory of Analytical Chemistry for Living Biosystems, Institute of Chemistry Chinese Academy of Sciences, Beijing Mass Spectrum Center, Beijing 100190, China; guanming@iccas.ac.cn (M.G.); linyu@iccas.ac.cn (Y.L.); zhangyy@iccas.ac.cn (Y.Z.); 3 Graduate School, University of Chinese Academy of Sciences, Beijing 100049, China

**Keywords:** triacylglycerols, phosphatidylcholine, ceramide, mass spectrometry, UPLC-ESI-QTOF MS

## Abstract

Background: The aim of this study was to characterize the disorder of lipid metabolism in hepatocellular carcinoma (HCC). HCC is a worldwide disease. The research into the disorder of lipid metabolism in HCC is very limited. Study of lipid metabolism in liver cancer tissue may have the potential to provide new insight into HCC mechanisms. Methods: A lipidomics study of HCC based on Ultra high performance liquid chromatography-electronic spray ionization-QTOF mass spectrometer (UPLC-ESI-QTOF MS) and Matrix assisted laser desorption ionization-fourier transform ion cyclotron resonance mass spectrometer (MALDI-FTICR MS) was performed. Results: Triacylglycerols (TAGs) with the number of double bond (DB) > 2 (except 56:5 and 56:4 TAG) were significantly down-regulated; conversely, others (except 52:2 TAG) were greatly up-regulated in HCC tissues. Moreover, the more serious the disease was, the higher the saturated TAG concentration and the lower the polyunsaturated TAG concentration were in HCC tissues. The phosphatidylcholine (PC), phosphatidylethanolamine (PE) and phosphatidylinositol (PI) were altered in a certain way. Sphingomyelin (SM) was up-regulated and ceramide (Cer) were down-regulated in HCC tissues. Conclusions: To our knowledge, this is the first such report showing a unique trend of TAG, PC, PE and PI. The use of polyunsaturated fatty acids, like eicosapentanoic and docosahexanoic acid, as supplementation, proposed for the treatment of Non-alcoholic steatohepatitis (NASH), may also be effective for the treatment of HCC.

## 1. Introduction

Liver cancer is one of ten leading cancer types of cancer death, and estimated new liver cancer cases and deaths were 33,190 and 23,000 respectively in the United States in 2014 [1]. Hepatocellular carcinoma (HCC) accounts for approximately 75% of all primary liver cancers, and chronic infection with either hepatitis C virus (HCV) or hepatitis B virus (HBV) leading to cirrhosis is the most common cause of HCC [2]. Although there are multiple therapeutic ways to treat HCC, such as liver resection, transplantation and ablation, the prognosis of HCC is still unsatisfying [3] and five-year survival rates are 17% in the United States (http://seer.cancer.gov/statfacts/html/livibd.html). A considerable number of gene expression studies have been undertaken targeted at understanding the progression of HCC [2]; however, the molecular-based mechanism that contributes to the pathogenesis of HCC is still poorly understood.

Lipid metabolism is one of the essential liver functions and the aberrant metabolism of liver has been reported to cause liver diseases such as inflammation and fibrosis [4]. Human liver pathogenesis of non-alcoholic fatty liver disease (NAFLD) is characterized by numerous changes in hepatic lipid composition and free fatty acid ratios [5,6]. High fat content and fructose consumption are the risk factors that may lead to NAFLD [7,8]. Significant expression changes of several phosphatidylcholines (PCs) and related enzymes were identified in the mouse livers of HBV infection and HBV-mediated regeneration defects [9]. Non-alcoholic steatohepatitis (NASH) represents a risk factor for the development of HCC, and analyses of liver samples from patients with NASH or NASH-related HCC show an elevated expression of the elongase ELOVL6 [10], which catalyzes the elongation of C16 to C18 fatty acids, and has been shown to promote NASH [11,12]. Patterson and co-workers investigated the disorder of metabolites and lipids in plasma of HCC patients. They found that HCC was associated with increased plasma levels of glycodeoxycholate, deoxycholate 3-sulfate, bilirubin, biliverdin, the fetal bile acids (7α-hydroxy-3-oxochol-4-en-24-oic acid and 3-oxochol-4,6-dien-24-oic acid), and reduced levels of lysophosphocholines (LPCs), lignoceric acid and nervonic acid [13]. Although the reason for these lipid molecules being either elevated or attenuated in plasma is not clear yet, lipidomics analyses may provide new biomarkers for HCC. Recently, a lipidomic analysis in a series of HCC cells was performed, and revealed that decreased palmitic acyl (C16:0)-containing glycerophospholipids were positively associated with metastatic abilities of HCC cells [14]. However, the research into the disorder of lipid metabolism in HCC tissues is very limited. The elucidations of the disorder of lipid metabolism in HCC tissues can significantly expanded our knowledge and engender new insights into HCC pathobiology [15].

Comprehensive study on the lipid changes in tissue at the molecular level is helpful to discover the role of lipid metabolism during tumor development and its mechanism, determine the key lipid metabolic pathways and related enzymes, and provide important basis for effective diagnosis and treatment. Therefore, in this study, we conducted lipidomic analyses in HCC and para-carcinoma tissues, to investigate the underlying molecular mechanism of HCC progression, using ultra performance liquid chromatography-electrospray ionization-quadrupole time-of-flight mass spectrometry (UPLC-ESI-QTOF MS) and matrix-assisted laser desorption ionization Fourier transform ion cyclotron resonance mass spectrometry (MALDI-FTICR MS).

## 2. Results

### 2.1. Profile Analysis of Lipids in Hepatocellular Carcinoma (HCC) and Para-Carcinoma Tissues by UPLC-ESI-QTOF MS

**Figure 1:**
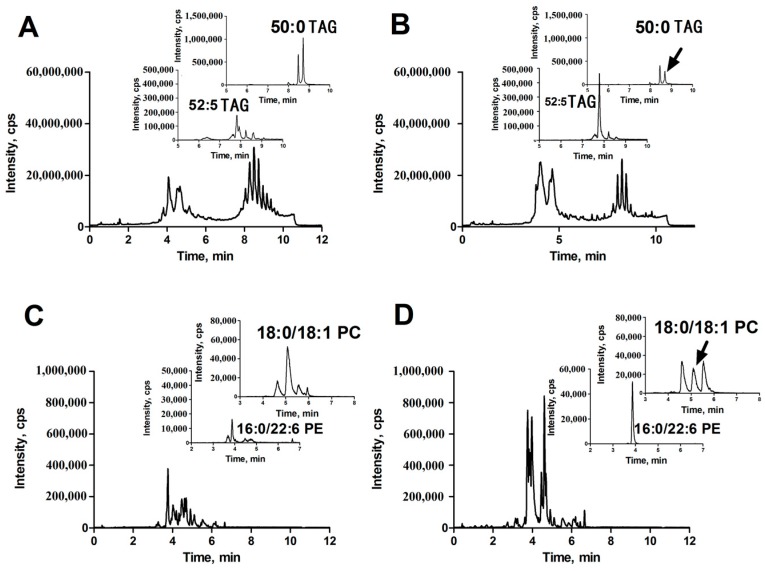
Representative total ion chromatograms (TICs) of lipid extracts obtained by UPLC-ESI (+)-QTOF MS (**A**,**B**) and UPLC-ESI (−)-QTOF MS (**C**,**D**). (**A**,**C**): The lipids were extract from HCC tissue; and (**B**,**D**): The lipids were extract from corresponding para-carcinoma tissue. The insets were extracted ion chromatograms (XICs) of indicated ions.

Using the UPLC-ESI-QTOF MS described above, we performed profile analysis of lipids in liver tissues in both positive and negative ion detection modes. The representative total ion chromatograms (TICs) of lipid extracts from HCC (Figure 1A,C) and corresponding para-carcinoma (Figure 1B,D) tissue obtained by UPLC-ESI (+)-QTOF MS (Figure 1A,B) and UPLC-ESI (−)-QTOF MS (Figure 1C,D) are shown in Figure 1. With the aid of the powerful analytical technique, many lipids were separated and identified. It was found that TAGs, glycerophospholipids and sphingolipids were the major components of liver lipids. The glycerophospholipids and sphingolipids were eluted between three and seven minutes and TAGs were eluted between seven to ten minutes. 

### 2.2. Orthogonal Partial Least Squared Discriminant Analysis (OPLS-DA) for Discrimination of HCC (HCC Group) and Para-Carcinoma Tissues (Para-Carcinoma Group)

**Figure 2:**
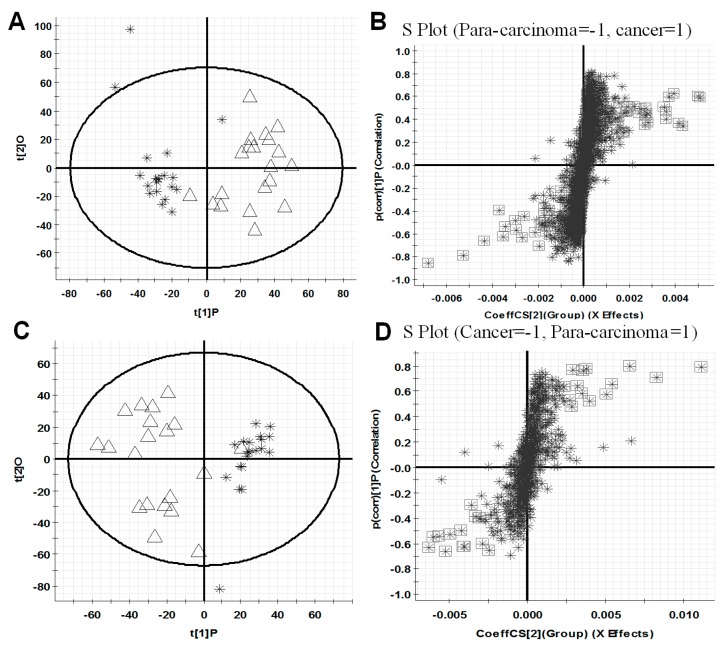
OPLS-DA score plots (**A**,**C**) for discrimination between HCC (∆, C) group and para-carcinoma (❋, P) group and S-plots (**B**,**D**) for selecting the potential markers based on the data from UPLC-ESI (+)-QTOF MS (**A**,**B**) or UPLC-ESI (−)-QTOF MS (**C**,**D**). The variables with VIP value > 4.0 were highlighted with black boxes (**B**,**D**). In (**A**,**C**), *X*-axis stands for the between group variation, and *Y*-axis stands for the within group variation. In (**B**,**D**), *X*-axis stands for the magnitude of viable change, and *Y*-axis stands for the confidence of viable change.

All observations acquired by the UPLC-ESI-QTOF MS-based lipidomics approach were integrated and co-analyzed using supervised OPLS-DA to differentiate HCC (HCC group) and para-carcinoma tissues (para-carcinaoma group). As presented in Figure 2A,C, a separation was observed between HCC group and para-carcinoma group, and similar results are also shown in Figure S1A,C illustrating the difference between the high TNM (tumor node metastasis) grade (III, IV) cancer tissues (C-H TNM) and low TNM grade (I, II) cancer tissues (C-L TNM), which suggested that lipid metabolic perturbations were evident and dependent on the pathological condition in the samples.

The variable importance in projection values (VIP) analysis in Markerlynx XS was further carried out to select variables as primary aberrant lipids for distinguishing the two groups. Based on the data, the corresponding S-plot, Figure 2B,D, are shown, respectively. In S-plot, each point represents a variable with specific retention time and mass-to-charge ratio (*m*/*z*) value. In our work, the variables with VIP value > 4.0 were highlighted with black boxes in Figure 2B,D. In summary, a total of 46 and 24 variables, listed in Table 1, were selected for distinguishing HCC group and para-carcinoma group, respectively, in positive ion and negative ion detection modes. These variables are primary aberrant lipids, greatly distinguish HCC group from para-carcinoma group, and therefore, merit further studies to identify their structures, which might provide new insights into the molecular mechanism of HCC progression. Similar results are also shown in Figure S1B,D, when differentiating the C-H TNM and C-L TNM. 

### 2.3. Identification of the Aberrant Lipids in HCC Group

The structures of these aberrant lipids were identified by database searching and were confirmed by standard lipids and/or MS/MS data. The protocol was similar to our previous work [16]. Here, a few examples were shown as below to further explain the detailed process.

**Figure 3:**
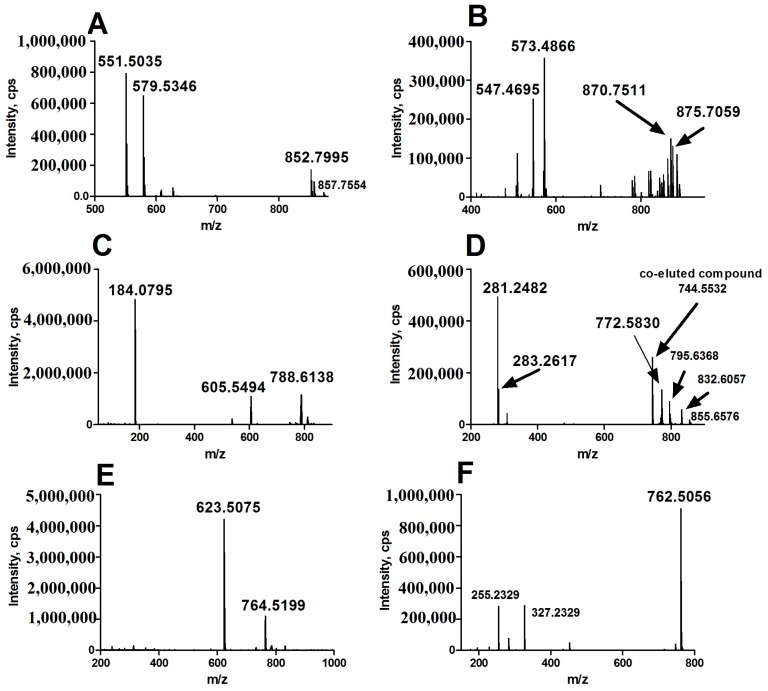
MS/MS data of representative aberrant lipids for structure identification. (**A**) lipid with RT at 8.72 in positive-ion detection mode; (**B**) lipid with RT at 7.80 min in positive-ion detection mode; (**C**) lipid with RT at 5.16 min in positive-ion detection mode; (**D**) lipid with RT at 5.16 min in negative-ion detection mode; (**E**) lipid with RT at 3.92 min in positive-ion detection mode; (**F**) lipid with RT at 3.92 min in negative-ion detection mode. *m*/*z*: mass-to-charge ratio.

In positive ion detection mode, two variables with retention times (RT) of 8.72 and 7.80 min and corresponding mass-to-charge ratios (*m*/*z*) of 852.7995 (up-regulated, shown in Figure 1A,B) and 870.7511 (down-regulated, shown in Figure 1A,B) were selected as two of primary changed lipids in HCC group. The database (LIPID MAPS) search results for the given *m*/*z* at 852.7995 and 870.7511 were ammonium adduct of 50:0 TAG and 52:5 TAG with a mass error −2.30 ppm and −3.92 ppm, respectively. The MS/MS spectrum with RT at 8.72 min in positive-ion detection mode (Figure 3A) showed serial of signals with *m*/*z* at 852.7995, 857.7554, 551.5035 and 579.5346, corresponding to the [50:0 TAG + NH_4_]^+^ (ammonium adduct), [50:0 TAG + Na]^+^ (sodium adduct), [50:0 TAG + NH_4_-17-C_16_H_32_O_2_]^+^ and [50:0 TAG + NH_4_-17-C_18_H_36_O_2_]+ (formed by neutral loss of NH_3_ and fatty acyl chain from ammonium adduct of TAG). This information and combinational restrictions arising from elemental composition allowed deducing the molecular species to be 18:0/16:0/16:0 TAG. Carefully investigating the MS/MS data at 7.80 min in positive-ion detection mode (Figure 3B), we could infer that the ions with *m*/*z* at 870.7511, 875.7059, 547.4695 and 573.4866 were corresponding to [52:5 TAG + NH_4_]^+^, [52:5 TAG + Na]^+^, [52:5 TAG + NH_4_-17-C_20_H_34_O_2_]^+^ and [52:5 TAG + NH_4_-17-C_18_H_32_O_2_]^+^ and the molecular species to be 20:3/14:0/18:2 TAG. Therefore, 50:0 TAG and 52:5 TAG were confirmed.

In positive ion detection mode, a variable with RT at 5.16 min and *m*/*z* at 788.6138 (up-regulated, shown in Figure 1C,D) was selected as one of the primary changed lipids in HCC group. The database (LIPID MAPS) search results for the given *m*/*z* at 784.5838 were 36:1 PC or 39:3 PE with a mass error −3.27 ppm. The MS/MS spectrum with RT at 5.16 min in positive-ion detection mode (Figure 3C) showed that an ion with *m*/*z* at 184.0795 (phosphorylcholine) was the main fragment ion. Based on our previous studies, it can be inferred that this ion (*m*/*z*: 788.6138) was likely a protonated PC. In negative ion detect mode, in the MS data with RT at 5.08 min, a quasimolecular ion with *m*/*z* at 832.6057 was shown. In the mobile phase, an ammonium formate was added as additive to enhance the ionization efficiency. Therefore, the ion with *m*/*z* at 832.6057 was likely the quasimolecular ion of [M + HCOO]^−^. The human metabolome database (HMDB) search results for the given *m*/*z* at 832.6057 were also showed that this compound was likely 36:1 PC with a mass error −1.93 ppm. The MS/MS data (Figure 3D) with RT at 5.08 min showed that two ions with *m*/*z* at 281.2482 (C_17_H_34_COO^−^) and at 283.2617 (C_17_H_36_COO^−^), corresponding to two fatty acyl chains linked to glycerol backbone in the structure of PC, were the main fragment ions. All together, we inferred that the variable with RT at 5.16 min was 18:0/18:1 PC.

In positive ion detection mode, a variable with RT at 3.92 min and *m*/*z* at 764.5199 (down-regulated, shown in Figure 1C,D) was also selected as one of the primary changed lipids in the HCC group. The database (LIPID MAPS) search results for the given *m*/*z* at 764.5199 were 35:6 PC or 36:6 PE with a mass error −3.38 ppm. The MS/MS spectrum with RT at 3.92 min in positive-ion detection mode (Figure 3E) showed that an ion with *m*/*z* at 623.5075 (neutral loss of phosphorylethanolamine) was the main fragment ion; it can be inferred that this ion (*m*/*z*: 764.5199) was likely a protonated phosphatidylethanolamine (PE). In negative ion detection mode, in the MS data with RT at 3.87 min, a quasimolecular ion with *m*/*z* at 762.5056 was shown. The database (HMDB) search results for the given *m*/*z* at 762.5056 also showed that this compound was likely deprotonated 36:6 PE with a mass error −3.05 ppm. The MS/MS data (Figure 3F) with RT at 3.87 min showed that two ions with *m*/*z* at 255.2329 (C_15_H_32_COO^−^) and at 327.2329 (C_21_H_32_COO^−^), corresponding to two fatty acyl chains linked to a glycerol backbone in the structure of PE, were the main fragment ions. All together, we inferred that the variable with RT at 3.92 min was 16:0/22:6 PE.

Similarly, we identified some other aberrant lipids. In total, 53 lipids were identified as major aberrant lipids in HCC group compared with the para-carcinoma group. More detailed information, including fold change (FC, the ratio of lipid level in HCC group versus para-carcinoma group), *p* value, etc. is listed in Table 1. Basically, the mainly identified aberrant lipids were TAGs, glycerophospholipids and sphingolipids. The variables simultaneously identified by both positive and negative ion detection modes showed a very consistent trend. For example, the FC of 16:0/22:6 PC were 0.58 and 0.54 in positive and negative ion detection modes, respectively, which further confirmed the reliability and accuracy of our MS-based lipidomics approach. 

**Figure 4:**
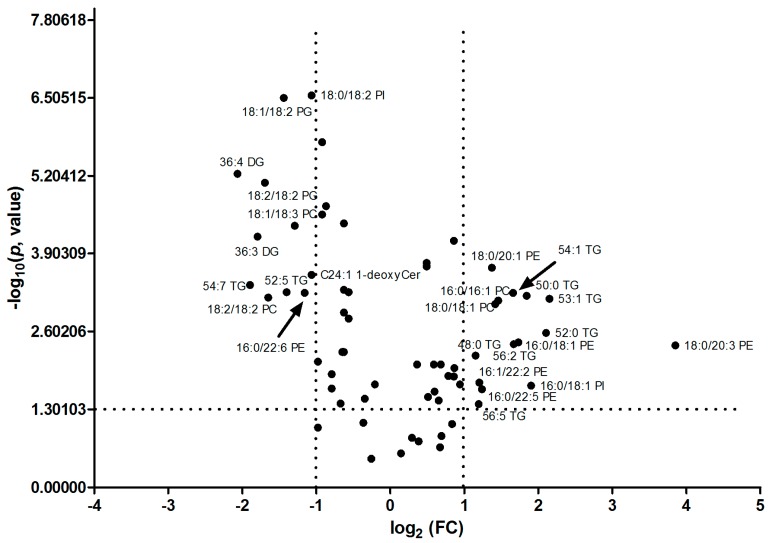
The volcano plot of aberrant lipids in hepatocellular carcinoma (C) vs. para-carcinoma (P) tissues. 26 specific lipids with fold change (FC) < 0.5 or >2.0 and *p* value < 0.05 were indicated in the volcano plot. FC: fold-change of C vs. P.

In addition, as revealed of FC in Table 1, it was found that TAGs with the number of double bonds more than 2 (except 56:5 and 56:4 TAG) were significantly down-regulated; however, TAGs with the number of double bonds equal to or less than 2 (except 52:2 TAG) were greatly up-regulated in HCC tissues. PC, PE and PI, with the number of double bonds equal to 0, 1 or 3, were significantly up-regulated, while PC, PE and PI (except 18:2/18:2 PI), with the number of double bonds equal to 2, 4 or 6, were significantly down-regulated. SM was up-regulated and Cer and PG were down-regulated in HCC tissues. The volcano plot of individual lipid in hepatocellular carcinoma vs. para-carcinoma tissues was plot, shown in Figure 4. 26 specific lipids with FC < 0.5 or >2.0 and *p* value < 0.05 were indicated. 

Further, the major aberrant lipids in the C-H TNM compared with C-L TNM were also identified and listed in Table S1, which showed that TAGs with the number of double bonds more than 4 were down-regulated, and saturated TAGs and TAGs with 1 double bond were up-regulated, although the *p* value was not significant. 

### 2.4. Matrix Assisted Laser Desorption Ionization Mass Spectrometry Imaging (MALDI MSI) Analysis of Human Liver Cancer Tissue

**Figure 5:**
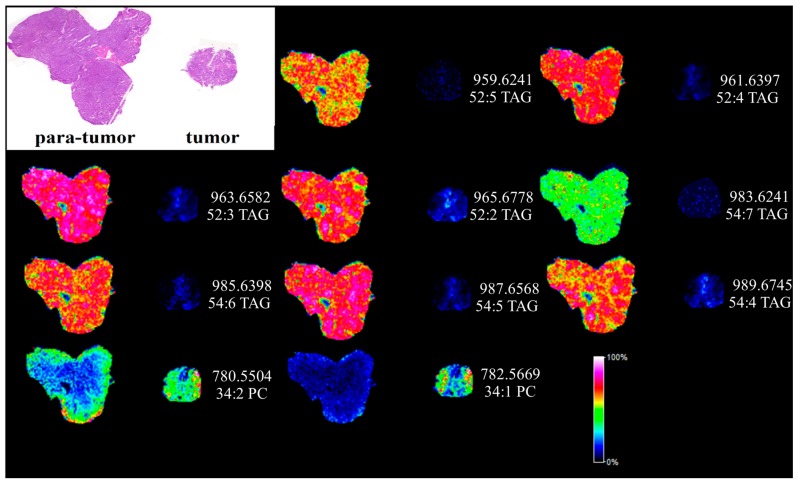
In situ MALDI imaging of lipids in human paracancerous and cancer tissue using AgNPs as matrix and the corresponding H&E staining image. The liver tissues were sectioned at 10 μm and the laser raster step size is 150 μm.

MALDI MSI has been widely applied for the label-free visualization of biological compounds from matrix coated tissue sections [17]. In order to directly visualize the dysfunction of lipids in HCC, silver nanoparticles (AgNPs)-based MALDI MSI was utilized and the results are shown in Figure 5. Hematoxylin-eosin (H&E) staining was performed to display the histological morphology of HCC and para-carcinoma tissues also shown in Figure 5. TAGs and PCs were imaged quite differently between the para-tumor liver tissue and the tumor tissues. The detected TAGs and PCs from MALDI MSI analysis are shown in Table S2. Eight highly unsaturated TAGs (two or more than two double bonds) were found extremely down regulated in the cancer section shown in Table S2. In contrast, 34:1 PC was found highly expressed in the liver cancer tissue section. The MALDI MSI images were quite consistent with the results obtained from UPLC-ESI-QTOF MS.

## 3. Discussion

Hepatocellular carcinoma (HCC) is a highly lethal cancer, with increasing worldwide incidence, that is mainly associated with chronic hepatitis B virus (HBV) and/or hepatitis C virus (HCV) infections. There are few effective treatments partly because the cell- and molecular-based mechanisms that contribute to the pathogenesis of this tumor type are poorly understood. In this study, 20 HCC tissues (16 HBV-related HCC, one HCV-related HCC, one HBV and HCV-related HCC, and two HCC without HBV and HCV infection) and their corresponding para-carcinoma tissues were collected for the study of disease mechanism.

Lipids are one of main components of cells and play essential roles in many cellular processes, such as cell growth, proliferation, differentiation, survival, apoptosis and drug resistance [18,19]. Recently, methods developed for lipid analysis, in particularly the method of electrospray ionization mass spectrometry (ESI-MS), realized the rapid and sensitive analysis of the majority of lipids in one analysis. For example, Giavalisco et al. combined an ultra-performance liquid chromatography (UPLC) with a high resolution MS and all-ion MS/MS for the semi-quantitative analysis of lipids extracted from *Arabidopsis thaliana* leaf, mainly including PC, PE, PG, PI, PS, etc. [20]. MS-based lipidomic analyses have significantly expanded our knowledge related to human physiology and pathology [15,21,22]. For example, plasma LPCs levels were decreased in colorectal cancer (CRC) patients, and have been identified as the potential diagnostic biomarker for CRC by ESI-MS [21,22]. Patterson and co-workers also investigated the disorder of metabolites and lipids in plasma of HCC patients [13]. Although the reason for these lipid molecules being either elevated or attenuated in plasma or liver tissue is not clear yet, lipidomic analyses have the potential to provide a new insight for the mechanism of HCC progression.

In this study, we conducted lipidomic analyses in HCC and para-carcinoma tissues using UPLC-ESI-QTOF MS and MALDI-FTICR MS. Multivariate data analysis was applied for investigating the aberrant lipid metabolism in hepatocellular carcinoma. High-resolution mass spectrometry and its corresponding MS/MS data were combined to accurately identify the structure of these aberrant lipids. Lipid metabolism in HCC was also concerned by Van Thiel DH et al. [23]. Interestingly, in our work, it was found that TAGs with the number of double bonds more than 2 (except 56:5 and 56:4 TAG) were significantly down-regulated; however, TAGs with the number of double bonds equal to or less than 2 (except 52:2 TAG) were greatly up-regulated in HCC tissues. Similar results were also obtained from the MADLI MSI analysis. Moreover, results showed that the more serious the disease was, the higher the saturated TAG concentration and the lower the polyunsaturated TAG concentration were in HCC tissues. PC, PE and PI, with the number of double bonds equal to 0, 1 or 3, were significantly up-regulated, while PC, PE and PI (except 18:2/18:2 PI), with the number of double bonds equal to 2, 4 or 6, significantly down-regulated. The MALDI MSI imaging of 34:1 PC also showed high expression in the tumor section. SM was up-regulated and Cer and PG were down-regulated in HCC tissues. To our knowledge, this is the first such report showing the unique trend of TAG, PC, PE and PI in HCC tissues, although impaired TAG metabolism associated with metabolic syndrome has been observed [24].

In the human body, high levels of plasma TAGs have been linked to cardiovascular disease [25,26,27]. The level of TAG has already been used as an index for evaluating metabolic syndromes by WHO [28]. There was a trend for increased saturated fatty acids (SFAs) and monounsaturated fatty acids (MUFAs), and a significant decreased polyunsaturated fatty acid (PUFA) associated with diacylglycerol (DAG) and TAG in NAFLD and NASH, compared with controls [5]. In our work, two DAGs and seven TAGs with the number of double bonds more than 2 were significantly decreased, while 14 TAGs with the number of double bonds equal to or less than 2 were greatly increased in HCC tissues compared with para-carcinoma tissues. Similar results were also shown in the images of polyunsaturated TAGs in MALDI MSI. Although the reason for the inhomogeneous change of TAGs was not clear, these changes could play a role in the pathogenesis of HCC.

Glycerophospholipids are major components of the cell membrane, and they have important roles during liver regeneration and lipid metabolism. The plasma lipidomics study revealed significantly increased total plasma monounsaturated fatty acids driven by palmitoleic (16:1 n7) and oleic (18:1 n9) acids content (*p* < 0.01 for both acids in both NAFLD and NASH). In addition, linoleic acid (18:2 n6) was decreased (*p* < 0.05), with a concomitant increase in linolenic (18:3 n6) and dihomolinolenic (20:3 n6) acids in both NAFLD and NASH (*p* < 0.001 for most lipid classes) [6]. In our work, coincidentally, PCs containing 16:1 and 18:1 fatty acyl chain were significantly increased, PCs containing 18:2 fatty acyl chain were significantly decreased, and PCs containing 18:3 and 20:3 fatty acyl chain were correspondingly significantly increased in HCC tissues compared with para-carcinoma tissues. In addition, both 16:0/22:6 PC and 18:0/22:6 PC were significantly decreased. The n-3 fatty acid (22:6 fatty acid) has profound anti-inflammatory, antiproliferative, immunomodulatory and metabolic effects [29,30,31,32], and the use of eicosapentanoic (20:5 n-3) and docosahexanoic (22:6 n-3) acid supplementation for the treatment of NASH has already been proposed [5]. Based on our results, this strategy may also be effective for the treatment of HCC.

Several studies have demonstrated that 70% of PC synthesized in the liver is mediated through the cytidine 5′-diphosphocholine (CDP)-choline pathway, while the remaining 30% is produced by the phosphatidylethanolamine *N*-methyltransferase pathway [33]. In our work, we found that PC and PE, with the number of double bonds equal to 0, 1 or 3, were significantly up-regulated, while PC and PE, with the number of double bonds equal to 2, 4 or 6, were significantly down-regulated, suggesting a strong association between PC and PE.

We found that Cer was decreased in HCC tissue. Cer is a well-known second messenger for apoptosis, senescence or autophagy [34]. Cer can be generated via hydrolysis of cell membrane SM by sphingomyelinases. We also found that SM was increased in HCC tissue. Taken together, the reduction of Cer in HCC tissue may decrease the cancer cell apoptosis, and the sphingomyelinase enzyme may be related to HCC.

## 4. Materials and Methods

### 4.1. Materials

HPLC-grade methanol (MeOH), acetonitrile (CH_3_CN), isopropanol (IPA), formic acid as well as ammonium formate were purchased from Sigma or Fisher Scientific (Pittsburgh, PA, USA). AgNO_3_ and polyvinyl pyrrolidone were purchased from Sigma-Aldrich (St. Louis, MO, USA). Ultrapure water was from Milli-Q purification system (Millipore Corporation, Burlington, MA, USA). Standard lipids, including l-α-phosphatidic acid (PA, Egg, Chicken), 1-palmitoyl-2-oleoyl-sn-glycero-3-phosphocholine (16:0/18:1 PC), 1-palmitoyl-2-arachidonoyl-sn-glycero-3-phosphocholine (16:0/20:4 PC), l-α-phosphatidylethanolamine (PE, egg, Chicken), l-α-phosphatidylglycerol (PG, egg, Chicken), l-α-phosphatidylinositol (PI, soy), l-α-phosphatidylserine (PS, brain, Porcine), *N*-heptadecanoyl-d-erythro-sphingosine (17:0 Cer), *N*-palmitoyl-d-erythro-sphingosine (16:0 Cer), *N*-palmitoyl-d-erythro-sphingosylphosphorylcholine (16:0 SM), *N*-oleoyl-d-erythro-sphingosylphosphorylcholine (18:1 SM), *N*-(octadecanoyl)-sphing-4-enine-1-phosphocholine (18:0 SM, brain) and 1,3(d5)-diheptadecanoyl-2-heptadecenoyl-glycerol (d5-(17:0-17:1-17:0) TAG) were purchased from Avanti Polar Lipids (Birmingham, AL, USA). The sphingoid base of Cer and SM were all d18:1. These lipids were used for assistance of qualitative analysis and as quality control of analysis. All of the above materials were used as received without further purification. The AgNPs were synthesized using polyvinyl pyrrolidone (PVP, MW = 55,000) as ligands based on the method of Xia [35].

### 4.2. Tissue Samples

The HCC and para-carcinoma tissues were obtained from 20 patients (54.8 ± 12.0 years old) undergoing cancer resection at Peking University People’s Hospital. Ethical approval of the present study was obtained from the ethical committee of Peking University People’s Hospital (Beijing, China). The methods were carried out in accordance with the approved guidelines. Tissue samples were collected in tubes, quickly frozen using liquid nitrogen and stored at −80 °C until use. All the patients were informed and the consents were obtained. 

The demographics and clinical measurements of patients were displayed in Table 2. They are comprised of 15 males (55.9 ± 10.8 years old) and 5 females (51.2 ± 16.0 years old). Among them, 16 had HBV-related HCC, 1 had HCV-related HCC, 1 had HBV and HCV-related HCC, and 2 had HCC without HBV and HCV infection. The TNM stage was classified according to UICC/AJCC, 2010. The median alpha fetal protein (AFP) concentration was 421.9 (range: 2.5–1210.0, mean ± SD: 421.9 ± 545.5) ng/mL, and 11 HCC patients had AFP values above a threshold of 20 ng/mL. In addition, there were 9 patients with vessel invasion. 

### 4.3. Lipids Extraction

Tissue samples were homogenized using Tissuelyser-24 (Shanghai Jingxing Experimental Technology, Shanghai, China). The methanol method that we have developed was used to extract lipids [36]. In brief, 50 μL of homogenate (water solution containing protease inhibitor, 10.0 mg of the tissue) from each sample was added to 950 μL of MeOH for lipids extraction. After vortexing and centrifugation (10,000× *g*, 5 min, room temperature), 300 μL of the supernatant was used for analysis by UPLC-ESI-QTOF mass spectrometer. 2 μL of the supernatant or 2 μL of 10× dilution were loaded into mass spectrometer for negative-ion or positive-ion lipids analysis, respectively.

### 4.4. Tissue Sectioning

The frozen HCC and para-carcinoma tissue were sectioned at 10 μm thickness using a Leica CM1950 cryostat (Leica Microsystems GmbH, Wetzlar, Germany) at −18 °C and mounted onto one indium tin oxide (ITO) coated glass slides ((Type I) 1.1 mm/100 ea, HST Inc., Newark, NJ, USA). The glass slide was then drying for approximately 1hr using a vacuum desiccator. The AgNPs MALDI matrix (2 mg/mL) was sprayed on the liver tissues by a homemade electrospray-based matrix deposition device described in the previous work [17]. The slide was dried by the vacuum desiccator for approximately 1hr again and then was used for MALDI MSI analysis.

### 4.5. UPLC-ESI-QTOF Mass Spectrometer

Lipids profile analysis was performed using Xevo G2 QTOF mass spectrometer (Waters). Both the nebulizer and desolvation gases were nitrogen. Typical operating parameters were set as follows: the capillary voltage was 3.0 kV or −3.0 kV in positive or negative ion mode, respectively; the sampling cone voltage was 40 V, desolvation gas temperature was 500 °C, the source temperature was set at 150 °C, nebulization gas flow was 40 L/h, and desolvation gas flow was 400 L/h. Data were collected between *m*/*z* 50 and 1200. Multiplexed data acquisition (MSE), a kind of data independent acquisition mode, was performed for simultaneously acquiring MS and MS/MS data with high resolution and high accuracy. The lock spray was used to ensure reproducibility and accuracy. Leucine enkephalin (200 pg/mL) was used as lock mass in ESI (−) (*m*/*z* 554.2620) and ESI (+) (*m*/*z* 556.2766). The lock spray frequency was set to 5 s, and the lock mass data were averaged over a total of 10 scans.

Samples were loaded through a LC system (I-class Acquity ultra performance liquid chromatography, Waters, Milford, MA, USA) with an auto sampler. The mobile phase A was IPA/CH_3_CN/formic acid (90:10:0.1, *v*/*v*/*v*) containing 10 mM ammonium formate; the mobile phase B was CH_3_CN/water/formic acid (70:30:0.1, *v*/*v*/*v*) containing 10 mM ammonium formate. A HSS T3 column (1.8 μm, 2.1 mm ID × 100 mm, Waters) was used for the separation of lipids. The column was maintained at 55 °C. The UPLC separations were 12 min/sample using the following scheme: (1) 0 min, 60% B; (2) 10 min, 1% B; (3) 12 min, 60% B. All the changes were linear, and the flow rate was set at 400 μL/min.

### 4.6. MALDI-FTICR Mass Spectrometry Imaging

MALDI-FTICR mass spectrometry imaging was performed with a Bruker SolariX mass spectrometer equipped with a 9.4 T superconducting magnet (SolariX^®^; Bruker, Bremen, Germany) using AgNPs as matrix. The concentration of AgNPs is 2 mg/mL dissolved in CH_3_CN:H_2_O (50:50, *v*/*v*). Positive ion mode was applied in all the experiments. Mass calibrations were performed externally using sodium trifluoroacetate (NaTFA, Sofia, Bulgaria). For MALDI-FTICR MS imaging, the parameters of MS instrument was the same with the previous work [17].

### 4.7. Data Processing

Data obtained by QTOF MS were processed by Markerlynx XS (Waters). As described in detail previously [16], orthogonal partial least square-discriminant analysis (OPLS-DA) was used for discrimination of HCC (HCC group) and para-carcinoma tissues (Para-carcinoma group). Discriminating variables, that is, aberrant lipids, were selected according to variable importance in projection values (VIP) > 4.0. The aberrant lipids were identified by searching local database established by Waters or free online databases, including Lipidmaps (http://www.lipidmaps.org/) and the human metabolome database (HMDB) (http://www.hmdb.ca), to precisely match the accurate molecular ion obtained by MS (mass error < 5 ppm). In addition, standard lipids were also used for assistance of identification of the aberrant lipids. Moreover, the fragment ions in MS/MS data obtained by collision induced dissociation (CID) were further utilized for confirmation of the structure of the aberrant lipids.

MALDI-FTICR MS Data were processed using DataAnalysis 4.0 (Bruker Daltonics, Billerica, MA, USA), FlexImaging 3.0 sofware (Bruker Daltonics, Billerica, MA, USA) and Isotope Parttern (Bruker Daltonics, Billerica, MA, USA). The lipids were identified by searching from HMDB as described before.

## 5. Conclusions

In conclusion, we have undertaken a liver lipidomics study of HCC, in which HCC and para-carcinoma tissue were used. Interestingly, it was found that TAGs with the number of double bonds more than 2 (except 56:5 and 56:4 TAG) were significantly down-regulated, and similar results were confirmed with MALDI MSI analyses; however, TAGs with the number of double bonds equal to or less than 2 (except 52:2 TG) were greatly up-regulated in HCC tissues. PC, PE and PI, with the number of double bonds equal to 0, 1 or 3, were significantly up-regulated, while PC, PE and PI (except 18:2/18:2 PI), with the number of double bonds equal to 2, 4 or 6, were significantly down-regulated. MALDI MSI imaging of 34:1 PC also showed high expression in the tumor section. SM was up-regulated and Cer and PG were down-regulated in HCC tissues. All these molecular changes in the liver of HCC patients provide new insights into the pathobiology of the disease. Further studies, such as induction of lipid dysfunction in normal liver tissue, lipid function, and the effect of lipid on the diagnostic and prognosis, are definitely needed.

## Figures and Tables

**Table 1:** The mainly changed lipids in HCC (C) compared with para-carcinoma tissue (P). RT, retention time; TAG, Triacylglycerol; FC, fold change; PG, Phosphatidylglycerol; Dark grey: FC > 1; Light grey: FC < 1.

Aberrant Lipid	RT (min)	Quasimolecular Ion	*m*/*z*		Error (ppm)	*p* Value	FC
			Detected	Theoretical			(C/P)
46:1 TAG	7.94	[M + NH_4_]^+^	794.7217	794.7232	−1.91	0.2163	1.60
46:0 TAG	8.19	[M + NH_4_]^+^	796.7369	796.7389	−2.47	0.0890	1.79
48:2 TAG	7.98	[M + NH_4_]^+^	820.7380	820.7389	−1.10	0.1409	1.62
48:1 TAG	8.21	[M + NH_4_]^+^	822.7521	822.7545	−2.93	0.0365	1.58
48:0 TAG	8.45	[M + NH_4_]^+^	824.7680	824.7702	−2.62	0.0065	2.23
50:1 TAG	8.47	[M + NH_4_]^+^	850.7843	850.7858	−1.78	0.0092	1.29
50:0 TAG	8.72	[M + NH_4_]^+^	852.7995	852.8015	−2.30	0.0006	3.60
51:1 TAG	8.60	[M + NH_4_]^+^	864.7989	864.8015	−3.01	0.0143	1.82
52:5 TAG	7.80	[M + NH_4_]^+^	870.7511	870.7545	−3.92	0.0006	0.38
52:4 TAG	8.03	[M + NH_4_]^+^	872.7698	872.7702	−0.42	0.0000	0.53
52:3 TAG	8.26	[M + NH_4_]^+^	874.7859	874.7858	0.10	0.0339	0.79
52:2 TAG	8.49	[M + NH_4_]^+^	876.8004	876.8015	−1.21	0.0193	0.87
52:1 TAG	8.73	[M + NH_4_]^+^	878.8148	878.8171	−2.63	0.0001	1.82
52:0 TAG	8.95	[M + NH_4_]^+^	880.8298	880.8328	−3.36	0.0027	4.32
53:1 TAG	8.83	[M + NH_4_]^+^	892.8300	892.8328	−3.14	0.0007	4.46
54:7 TAG	7.58	[M + NH_4_]^+^	894.7510	894.7545	−3.93	0.0004	0.27
54:6 TAG	7.83	[M + NH_4_]^+^	896.7674	896.7702	−3.12	0.0081	0.51
54:5 TAG	8.06	[M + NH_4_]^+^	898.7831	898.7858	−3.02	0.0000	0.53
54:4 TAG	8.29	[M + NH_4_]^+^	900.7993	900.8015	−2.40	0.0006	0.68
54:2 TAG	8.73	[M + NH_4_]^+^	904.8303	904.8328	−2.72	0.0090	1.51
54:1 TAG	8.96	[M + NH_4_]^+^	906.8451	906.8484	−3.65	0.0006	3.17
56:5 TAG	8.39	[M + NH_4_]^+^	926.8146	926.8171	−2.70	0.0413	2.30
56:4 TAG	8.57	[M + NH_4_]^+^	928.8298	928.8328	−3.23	0.0196	1.93
56:2 TAG	8.95	[M + NH_4_]^+^	932.8607	932.8641	−3.64	0.0042	3.19
36:4 DAG	4.99	[M + NH_4_]^+^	634.5394	634.5405	−1.73	0.0000	0.24
36:3 DAG	5.42	[M + NH_4_]^+^	636.5544	636.5561	−2.75	0.0001	0.29
24:1 SM	5.60	[M + H]^+^	813.6817	813.6844	−3.32	0.0141	1.73
C24:1 1-deoxy Cer	6.72	[M + H]^+^	632.6325	632.6340	−2.37	0.0003	0.48
22:0 Cer	6.20	[M + HCOO]^−^	666.6029	666.6042	−1.94	0.0005	0.65
23:0 Cer	6.44	[M + HCOO]^−^	680.6181	680.6198	−2.57	0.0000	0.55
24:0 Cer	6.65	[M + HCOO]^−^	694.6343	694.6355	−1.72	0.0012	0.65
16:0/16:1 PC	3.97	[M + H]^+^	732.5524	732.5538	−1.89	0.0008	2.76
	3.89	[M + HCOO]^−^	776.5434	776.5447	−1.68	0.0040	3.17
16:0/16:0 PC	4.46	[M + H]^+^	734.5680	734.5694	−1.95	0.0315	1.43
	4.40	[M + HCOO]^−^	778.5589	778.5604	−1.87	0.0047	1.55
16:0/18:2 PC	4.00	[M + HCOO]^−^	802.5597	802.5604	−0.82	0.0015	0.68
16:0/18:1 PC	4.45	[M + HCOO]^−^	804.5749	804.5760	−1.37	0.0002	1.41
16:0/20:4 PC	3.98	[M + H]^+^	782.5687	782.5694	−0.93	0.0854	0.78
16:0/20:3 PC	4.23	[M + H]^+^	784.5836	784.5851	−1.89	0.1719	1.31
16:0/22:6 PC	3.85	[M + H]^+^	806.5679	806.5694	−1.86	0.0228	0.58
	3.80	[M + HCOO]^−^	850.5585	850.5604	−2.18	0.0003	0.54
18:2/18:2 PC	3.73	[M + H]^+^	782.5675	782.5694	−2.47	0.0007	0.32
18:0/18:1 PC	5.16	[M + H]^+^	788.6138	788.6164	−3.27	0.0009	2.68
	5.08	[M + HCOO]^−^	832.6057	832.6073	−1.93	0.0001	2.63
18:0/20:4 PC	4.59	[M + H]^+^	810.5988	810.6007	−2.38	0.3424	0.84
18:0/20:3 PC	4.87	[M + H]^+^	812.6139	812.6164	−3.05	0.0105	1.83
	4.81	[M + HCOO]^−^	856.6053	856.6073	−2.34	0.0038	1.68
18:0/22:6 PC	4.44	[M + H]^+^	834.5988	834.6007	−2.31	0.0407	0.63
16:0/18:1 PE	3.89	[M − H]^−^	716.5218	716.5236	−2.48	0.0039	3.33
16:0/22:6 PE	3.92	[M + H]^+^	764.5199	764.5225	−3.38	0.0006	0.45
	3.87	[M − H]^−^	762.5056	762.5079	−3.05	0.0000	0.51
16:0/22:5 PE	4.21	[M + H]^+^	766.5391	766.5381	1.26	0.0236	2.37
	4.17	[M − H]^−^	764.5224	764.5236	−1.54	0.0133	1.68
16:0/20:4 PE	4.03	[M − H]^−^	738.5066	738.5079	−1.76	0.2755	1.11
16:1/22:2 PE	4.95	[M + H]^+^	770.5683	770.5694	−1.47	0.0182	2.31
	4.85	[M − H]^−^	768.5532	768.5549	−2.18	0.0092	1.61
18:1/22:5 PE	4.52	[M + H]^+^	792.5519	792.5538	−2.37	0.0131	0.58
18:0/18:2 PE	4.00	[M + H]^+^	742.5380	742.5392	−1.62	0.0000	0.65
18:0/18:1 PE	4.45	[M + H]^+^	744.5533	744.5549	−2.12	0.0002	1.41
18:0/20:3 PE	5.01	[M + H]^+^	768.5525	768.5549	−3.10	0.0043	14.48
18:0/20:1 PE	5.08	[M + H]^+^	772.5841	772.5862	−2.69	0.0002	2.60
18:1/22:5 PE	4.46	[M + H]^+^	790.5376	790.5392	−2.06	0.0056	0.65
18:2/18:2 PG	2.74	[M − H]^−^	769.5004	769.5025	−2.73	0.0000	0.31
18:1/18:2 PG	3.16	[M − H]^−^	771.5164	771.5182	−2.28	0.0000	0.37
18:1/18:1 PG	3.57	[M − H]^−^	773.5320	773.5338	−2.34	0.1034	0.51
16:0/18:2 PI	3.24	[M − H]^−^	833.5166	833.5186	−2.34	0.0056	0.64
16:0/18:1 PI	3.67	[M − H]^−^	835.5328	835.5342	−1.68	0.0205	3.75
18:2/18:2 PI	3.19	[M − H]^−^	857.5170	857.5186	−1.87	0.1499	1.23
18:0/18:2 PI	3.83	[M − H]^−^	861.5486	861.5499	−1.51	0.0000	0.48
18:0/20:3 PI	4.03	[M − H]^−^	887.5642	887.5655	−1.46	0.0256	1.52

**Table 2:** Demographics and clinical measurements of patients.

Factor		No.	%
Sex	Male	15	75
	Female	5	25
TNM stage	I, II	13	65
	III, IV	7	35
AFP level	<20 ng/mL	9	45
	>20 ng/mL	11	55
vessel invasion	Yes	9	45
	No	11	55

## Supplementary Materials

Supplementary materials can be found at www.mdpi.com/1422-0067/18/12/2550/s1.

## Abbreviations

HCC	Hepatocellular carcinoma
UPLC-ESI-QTOF MS	Ultra high performance liquid chromatography-electronic spray ionization-QTOF mass spectrometer
MALDI-FTICR MS	Matrix assisted laser desorption ionization-fourier transform ion cyclotron resonance mass spectrometer
TAG	Triacylglycerol
DB	Double bond
PC	Phosphatidylcholine
PE	Phosphoethanolamine
PI	Phosphatidylinositol
SM	Sphingomyelin
Cer	Ceramide
HCV	Hepatitis C virus
HBV	Hepatitis B virus
NAFLD	Non-alcoholic fatty liver disease
NASH	Non-alcoholic steatohepatitis
LPC	Lysophosphatidylcholine
OPLS-DA	Orthogonal partial least squared discriminant analysis
MALDI MSI	Matrix assisted laser desorption ionization mass spectrometry imaging
TIC	Total ion chromatogram
TNM	Tumor node metastasis
C-H TNM	High TNM grade (III, IV) cancer tissue
C-L TNM	Low TNM grade (I, II) cancer tissue
VIP	Variable importance in projection value
RT	Retention time
HMDB	Human Metabolome Database
FC	Fold change
PG	Phosphatidylglycerol
AgNPs	Silver nanoparticles
H&E	Hematoxylin-eosin staining
ESI-MS	Electrospray ionization mass spectrometry
UPLC	Ultra high performance liquid chromatography
PS	Phosphatidylserine
CRC	Colorectal cancer
SFA	Saturated fatty acid
MUFA	Monounsaturated fatty acid
PUFA	Polyunsaturated fatty acid
DAG	Diacylglycerol
MeOH	Methanol
CH_3_CN	Acetonitrile
IPA	Isopropanol
PA	Phosphatidic acid
PVP	Polyvinyl pyrrolidone
AFP	Alpha fetal protein
ITO	Indium tin oxide
MSE	Multiplexed data acquisition
NaTFA	Sodium trifluoroacetate
CID	Collision induced dissociation
