# Supplementary material for: Aberrant Lipid Metabolism in Hepatocellular Carcinoma Revealed by Liver Lipidomics
Source: Int J Mol Sci. 2017 Nov 28;18(12):2550. doi: 10.3390/ijms18122550 (PMC5751153; doi:10.3390/ijms18122550)
Supplement: Supplementary file 1 [file ijms-18-02550-s001.pdf]

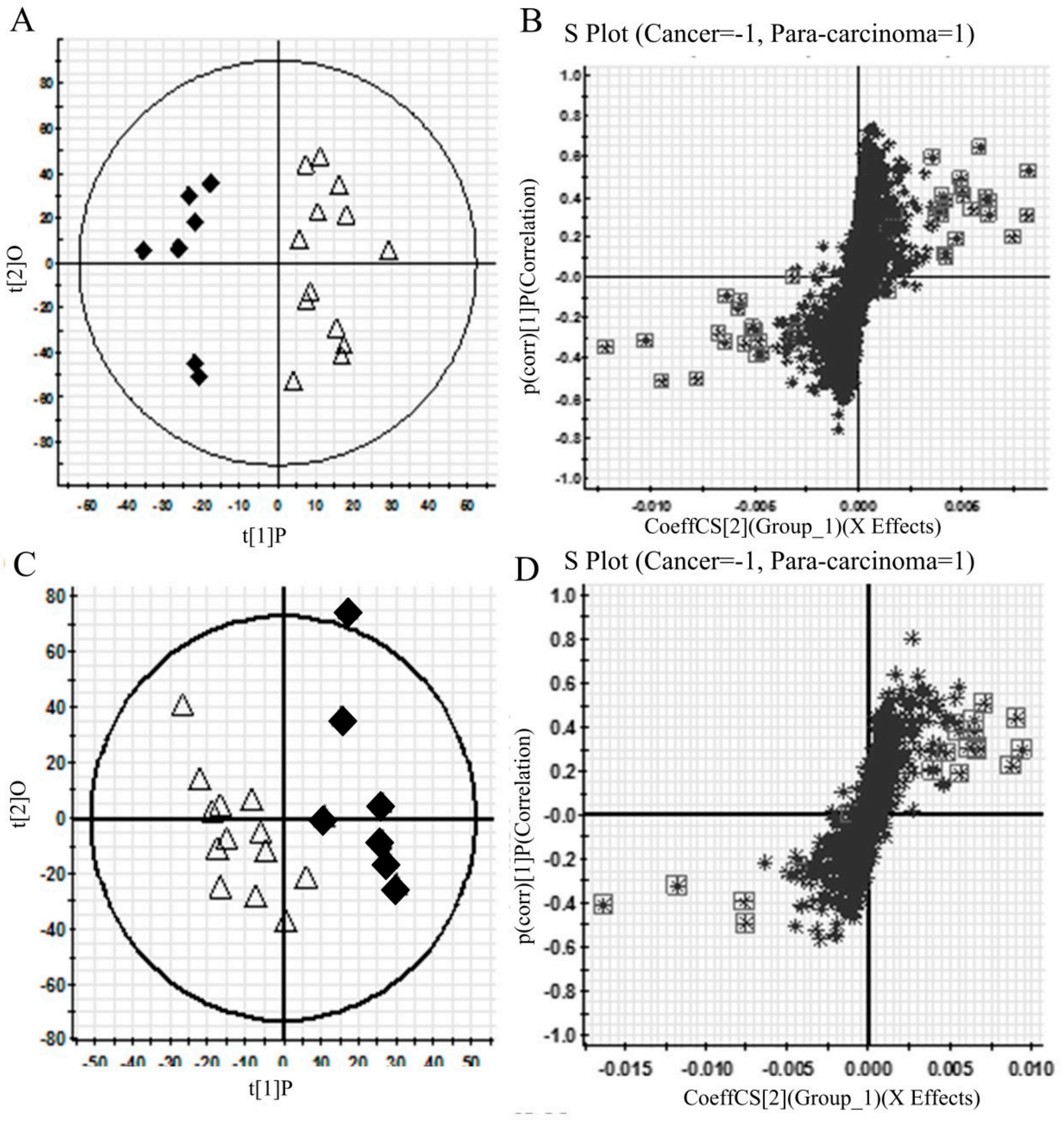

Figure S1. OPLS-DA score plots (A and C) and S-plots (B and D) based on the data from UPLC-ESI(+)-QTOF-MS (A and B) or UPLC-ESI(-)-QTOF-MS (C and D) for distinguishing discrimination between high TNM grade cancer tissue (♦, C-H TNM) group and low TNM grade cancer tissue (Δ, C-L TNM) group. The variables with VIP value >4.0 were highlighted with black boxes.

Table S1. The mainly changed lipids in high TNM grade cancer tissue (C-H TNM) compared with low TNM grade cancer tissue (C-L TNM). RT, retention time; FC, fold change.

Aberrant lipid	RT (min)	Quasimolecular ion	m/z		Mass error (ppm)	p value	FC (H/L)
			Detected	Exact			
44:0 TAG	7.90	$[M+NH_4]^+$	768.7051	768.7076	-3.21	0.2037	2.49
46:2 TAG	7.68	$[M+NH_4]^+$	792.7051	792.7076	-3.11	0.1923	2.64
46:1 TAG	7.94	$[M+NH_4]^+$	794.7217	794.7232	-1.91	0.3153	1.94
46:0 TAG	8.19	$[M+NH_4]^+$	796.7369	796.7389	-2.47	0.3374	2.22
48:3 TAG	7.73	$[M+NH_4]^+$	818.7206	818.7232	-3.19	0.1866	2.85
48:2 TAG	7.98	$[M+NH_4]^+$	820.738	820.7389	-1.10	0.3975	1.58
48:1 TAG	8.2134	$[M+NH_4]^+$	822.7521	822.7545	-2.93	0.6281	1.36
48:0 TAG	8.45	$[M+NH_4]^+$	824.768	824.7702	-2.62	0.5370	1.88
50:3 TAG	8.00	$[M+NH_4]^+$	846.7525	846.7545	-2.38	0.3852	0.97
50:1 TAG	8.47	$[M+NH_4]^+$	850.7843	850.7858	-1.78	0.9724	1.15
50:0 TAG	8.72	$[M+NH_4]^+$	852.7995	852.8015	-2.30	0.4665	1.61
51:3 TAG	8.15	$[M+NH_4]^+$	860.7667	860.7702	-4.02	0.1841	0.59
51:2 TAG	8.37	$[M+NH_4]^+$	862.7827	862.7858	-3.59	0.2372	0.73
51:1 TAG	8.60	$[M+NH_4]^+$	864.7989	864.8015	-3.01	0.2289	0.82
52:4 TAG	8.03	$[M+NH_4]^+$	872.7698	872.7702	-0.42	0.4253	0.73
52:3 TAG	8.26	$[M+NH_4]^+$	874.7859	874.7858	0.10	0.9210	0.91
52:1 TAG	8.73	$[M+NH_4]^+$	878.8148	878.8171	-2.63	0.6821	1.14
53:3 TAG	8.41	$[M+NH_4]^+$	888.7985	888.8015	-3.33	0.1892	0.60
53:2 TAG	8.61	$[M+NH_4]^+$	890.8137	890.8171	-3.83	0.2044	0.60
54:5 TAG	8.06	$[M+NH_4]^+$	898.7831	898.7858	-3.02	0.2380	0.84
54:4 TAG	8.29	$[M+NH_4]^+$	900.7993	900.8015	-2.40	0.4176	0.78
54:2 TAG	8.73	$[M+NH_4]^+$	904.8303	904.8328	-2.72	0.4292	1.26
56:7 TAG	8.01	$[M+NH_4]^+$	922.7828	922.7858	-3.27	0.2310	0.56
36:3 DAG	5.42	$[M+NH_4]^+$	636.5544	636.5561	-2.75	0.1682	0.17
16:0 SM	3.79	$[M+H]^+$	703.5733	703.5749	-2.20	0.3076	1.38
22:1 SM	5.61	$[M+H]^+$	787.6656	787.6688	-4.00	0.1963	0.53
16:0/16:1 PC	3.89	$[M+HCOO]^-$	776.5434	776.5447	-1.68	0.4672	1.38
16:0/16:0 PC	4.46	$[M+H]^+$	734.568	734.5694	-1.95	0.3970	0.69
	4.40	$[M+HCOO]^-$	778.5589	778.5604	-1.87	0.1030	0.69
16:0/18:2 PC	4.00	$[M+HCOO]^-$	802.5597	802.5604	-0.82	0.4277	0.85
16:0/18:1 PC	4.53	$[M+H]^+$	760.5852	760.5851	0.16	0.7794	0.89
16:0/20:4 PC	3.98	$[M+H]^+$	782.5687	782.5694	-0.93	0.7527	0.95
16:0/20:3 PC	4.23	$[M+H]^+$	784.5836	784.5851	-1.89	0.5581	0.63
	4.19	$[M+HCOO]^-$	828.5745	828.5760	-1.82	0.0663	0.78
16:0/20:2 PC	4.61	$[M+HCOO]^-$	830.5903	830.5917	-1.63	0.5007	1.15
16:0/22:6 PC	3.85	$[M+H]^+$	806.5679	806.5694	-1.86	0.2858	0.47
18:0/18:2 PC	4.69	$[M+H]^+$	786.6	786.6007	-0.93	0.1653	1.61
18:0/18:1 PC	5.16	$[M+H]^+$	788.6138	788.6164	-3.27	0.2677	1.52
	5.08	$[M+HCOO]^-$	832.6057	832.6073	-1.93	0.1564	1.50
18:0/20:4 PC	4.59	$[M+H]^+$	810.5988	810.6007	-2.38	0.3451	1.15
	4.54	$[M+HCOO]^-$	854.5896	854.5917	-2.41	0.1493	1.39
16:0/16:1 PE	3.97	$[M-H]^-$	688.4932	688.4923	1.34	0.2652	2.96
16:0/20:4 PE	4.03	$[M-H]^-$	738.5066	738.5079	-1.76	0.3455	1.19
16:0/22:6 PE	3.92	$[M+H]^+$	764.5199	764.5225	-3.38	0.2455	0.51
18:0/20:4 PE	4.67	$[M+H]^+$	768.5515	768.5538	-2.97	0.4815	1.08
	4.61	$[M-H]^-$	766.5377	766.5392	-1.99	0.3021	1.26
18:0/20:3 PE	5.01	$[M-H]^-$	768.5525	768.5549	-3.10	0.2199	2.12
18:1/22:5 PE	4.52	$[M+H]^+$	792.5519	792.5538	-2.37	0.2037	0.70
18:1/18:1 PG	3.57	$[M-H]^-$	773.5320	773.5338	-2.34	0.2818	1.78
16:0/16:1 PI	3.11	$[M-H]^-$	807.5006	807.5029	-2.85	0.1670	3.52
16:0/18:1 PI	3.67	$[M-H]^-$	835.5328	835.5342	-1.68	0.1775	2.11
18:0/18:2 PI	3.83	$[M-H]^-$	861.5486	861.5499	-1.51	0.1122	0.61
18:0/18:1 PI	4.23	$[M-H]^-$	863.5638	863.5655	-1.97	0.3759	1.36
18:0/20:4 PI	3.77	$[M-H]^-$	885.5484	885.5499	-1.69	0.8393	1.03
18:0/20:3 PI	4.03	$[M-H]^-$	887.5642	887.5655	-1.46	0.1041	0.59

Table S2. TAGs and PCs detected in human liver tissue section by MALDI FTICR MSI in positive ion mode using AgNPs as matrix.

Name	Measured m/z	Exact m/z	m/z error (ppm)	Ion type
34:2 PC	780.5504	780.5514	-1.4	$M+Na^+$
34:1 PC	782.5669	782.5670	-0.2	$M+Na^+$
52:5 TAG	959.6241	959.6252	-1.1	$M+Ag^+$
52:4 TAG	961.6397	961.6409	-1.2	$M+Ag^+$
52:3 TAG	963.6582	963.6565	1.8	$M+Ag^+$
52:2 TAG	965.6683	965.6722	-4.9	$M+Ag^+$
54:4 TAG	983.6241	983.6252	-1.1	$M+Ag^+$
54:7 TAG	985.6398	985.6409	-1.1	$M+Ag^+$
54:6 TAG	987.6568	987.6565	0.3	$M+Ag^+$
54:5 TAG	989.6745	989.6722	2.4	$M+Ag^+$
